# Pandemic disruptions in vaccine uptake in a low-income setting: a qualitative inquiry

**DOI:** 10.1186/s12919-023-00283-w

**Published:** 2023-11-09

**Authors:** Fauzia Aman Malik, Nazia Ahsan, Rawshan Jabeen, Osama Afzal, Alysha Siddiqi, Ayub Khan, Kathryn L. Hopkins, Abdul Momin Kazi

**Affiliations:** 1 Peter O’Donnell Jr. School of Public Health, UT Southwestern Medical Center, Dallas, Texas USA; 2 Yale School of Public Health, Yale University, New Haven, Connecticut USA; 3 Department of Paediatrics & Child Health, Aga Khan University, Karachi, Sindh Pakistan; 4 Sabin Vaccine Institute, Washington D.C, USA

**Keywords:** COVID-19 pandemic, Vaccine hesitancy, Routine childhood immunization, COVID-19 vaccines, Immunization systems, Vaccine communication strategy, Resilient immunization system, LMIC

## Abstract

**Background:**

While the COVID-19 pandemic has exposed the vulnerabilities of immunization delivery systems globally, the devastating impact of the pandemic on immunization delivery is most pronounced in low and middle-income countries like Pakistan. We conducted a qualitative study to capture the views and experiences of parents and healthcare workers (HWs) and assess the impact of the COVID-19 pandemic on childhood routine immunization (CRI) and COVID-19 vaccination in Pakistan.

**Methods:**

We used a qualitative research design with a purposive sampling approach. Semi-structured interviews (via telephone) and focus group discussions (via Zoom) were conducted with parents/child caregivers and HWs, respectively. All qualitative interviews were conducted between February and July 2021 from three sites (two urban and one rural) in Sindh, Pakistan. Interviews were audio-recorded, transcribed, and coded for a team-based thematic analysis.

**Results:**

Overall, most parents and HWs indicated a strong trust in the benefits of CRI; nonetheless, a substantial disruption in the delivery and uptake of these services was also reported. The barriers towards CRI included closed vaccination centers, drastic reduction in outreach programs, lack of information for parents/child caregivers on vaccine availability, fear in the community regarding vaccine safety, limited vaccine supply, and a lack of healthcare staff. For COVID-19 vaccines, challenges cited included skepticism about the reality of the pandemic and confusion over COVID-19 vaccines due to conflicting (or mis-or-dis) information. Both participant groups showed a willingness to integrate COVID-19 vaccination into Pakistan’s Expanded Program for Immunization if required in the future.

**Conclusion:**

During the COVID-19 pandemic, disruptions of regular immunization delivery in Pakistan were not due to parental unwillingness to vaccinate, but rather to social and logistical challenges caused by a rapidly changing context and difficulties in providing vaccination services safely. Barriers to vaccine access and concerns about COVID-19 exposure during clinic visits also contributed to uncertainty regarding immunization services early in the pandemic. For catchup campaigns and future pandemics, more than focusing interventions on persuading people, strategic approaches to building resilience through system-based interventions, such as investing in surge capacity in the immunization workforce to bounce back quickly after the first shock are required.

## Introduction

Immunization is a powerful public health tool used to protect people, particularly children, from life-threatening diseases and save millions of lives each year [1]. The importance of immunization became particularly apparent during the COVID-19 pandemic when countries around the world implemented quarantine measures to reduce the spread of the COVID-19 virus. While these safety measures mitigated the danger of COVID-19, they also had a detrimental effect on the provision of all basic healthcare services, including routine childhood immunizations (CRI) [2]. In May 2020, the World Health Organization (WHO) estimated there were 80 million children younger than one year of age at risk of missing life-saving vaccinations [3]. By July 2022, WHO and UNICEF reported that the pandemic had led to the longest sustained decline of CRI in 30 years: in 2021, 25 million children missed their routine vaccinations (18 million of whom were "zero-dose" children who had not received a single vaccine dose) for a 37% increase from 2019 to 2021 [4, 5]. Despite international collaboration and efforts to improve vaccination rates among children, studies illustrate a decline in vaccination coverage across all milestone age groups, except for vaccines administered at birth in hospitals, such as Hepatitis B [6]. For the first time in almost three decades, there was a significant decrease in the coverage of the diphtheria, pertussis, and tetanus (DPT3) vaccine dose [7]. Now, countries around the world are seeing outbreaks of previously controlled diseases like measles, rubella, tuberculosis, and rotavirus: even new mutated strains of polio have reemerged [2].

In Pakistan, childhood routine vaccinations for diseases such as DTP, polio, measles, and hepatitis B, are accessible through Primary Health Care (PHC) facilities and regular immunization clinics. Children are also reached through recurring immunization efforts, including door-to-door outreach programs. In the case of COVID-19 vaccination, distribution began with healthcare workers and high-risk groups before being extended to the broader public. This was accomplished with the assistance of the government and other partners, including non-governmental organizations (NGOs) and civil society organizations (CSOs), as well as additional community-based outreach programs [8]. In Pakistan, the network of CSOs is critical in the provision of vaccinations. The Pakistan CSOs Coalition for Health and Immunization (PCCHI) brings together around 80 organizations to improve health and immunization through community mobilization, research, advocacy and awareness, demand generation, monitoring, and service delivery [9, 10]. Despite these efforts, increasing coverage of CRI in low- and middle-income countries (LMICs) like Pakistan remains challenging (6). For example, Pakistan ranks among the top 10 countries for the number of under-immunized and zero-dose children. Based on the 2017–2018 National Demographic and Health Survey (DHS), vaccination rates across Pakistan remain suboptimal, with only 66% of children aged 12–23 months fully vaccinated [11]. The primary factors contributing to such low vaccination rates include missed vaccination opportunities, scheduling challenges, system breakdowns, supply chain obstacles, and parental vaccine hesitancy [12]. This leaves wide gaps for continuous polio transmission, significant measles outbreaks, and thousands of deaths from vaccine-preventable diseases [13].

When the COVID-19 pandemic began to overburden healthcare systems in early 2020, governments were forced to take severe steps, such as diverting resources from immunization campaigns to emergency COVID-19 response efforts [14]. To control the pandemic, the Government of Pakistan also implemented lockdowns and other social distancing measures recommended by the WHO. Although these prevention measures mitigated the threat of COVID-19, they inevitably led to the partial or complete closure of vaccination clinics across the country. These disruptions also altered the public’s health-seeking behavior, leaving large numbers of unvaccinated children susceptible to infections [15].

While the negative impact of the COVID-19 pandemic on immunization programs is clear, most research related to this topic is quantitative, limiting our understanding of the social and behavioral determinants of vaccine-related decision-making and behavior during the pandemic. To fill this gap in understanding, we conducted a qualitative study to assess the impact of the pandemic on CRI in Pakistan through the views and experiences of parents/child caregivers and healthcare workers (HWs). We also assessed respondents’ perspectives on the uptake of COVID-19 vaccines among adults and potentially children to identify challenges and barriers to immunization while considering system strengths and weaknesses and exploring the possibility of incorporating COVID-19 vaccines in Pakistan’s Expanded Program for Immunization (EPI).

## Methods

### Study design, study setting, and participants.

An exploratory qualitative research design was employed with a purposive sampling approach. Semi-structured in-depth interviews (IDIs) were conducted with parents/caregivers of children less than one year of age. Focus group discussions (FGDs) were held with the HWs (doctors, nurses, pharmacists, Lady Health Visitors (LHVs), Vaccinators, Lady Health Workers (LHWs), and Community Health Workers (CHWs) in Karachi and Matiari who worked in the selected centers and provided verbal informed consent. All three centers included in the study provided routine immunization as per the recommendation of the government EPI program. Data captured included their views and experiences of engagement with CRI during the pandemic and newly available COVID-19 vaccines for adults.

At the time of the study, COVID-19 vaccine trials for children were still underway; therefore, COVID-19 vaccines were only available and administered to adults. Study participants, both HWs and parents, were recruited from three sites, two in Karachi 1) Community Health Center (CHC) vaccination clinic at the Aga Khan University Hospital 2) Ali Akbar Shah Colony, Community Health Center) and one in Matiari (rural Sindh), located 185 km north of Karachi City. In Matiari, 95% of the population relies on agriculture for income and poverty affects 58.46% of the population. This is due to factors like inflation, unemployment, low crop prices, water scarcity, education gaps, weak infrastructure, limited credit access, and low rates of women's employment and education [16]. The Ali Akbar Shah site served as the Primary Healthcare Center (PHC) in the Health Demographic Surveillance System (HDSS) of The Aga Khan University, located in a peri-urban area of Karachi. The most common occupation in this area is fishing, and this area has a high poverty rate and literacy gaps [17]. The second urban site established tertiary care with the main center of the Cosmopolitan city, Karachi.

### Online data collection procedures

Due to social distancing and lockdowns, all data was collected via Zoom and telephone. Before the interviews, all participants provided verbal informed consent. A member of the research team introduced participants to and guided them through the Zoom platform and telephone conference calls. Trained qualitative researchers conducted interviews in Urdu and Sindhi languages. Audio recordings from the interviews were first transcribed in the language they were conducted in; these transcriptions were later translated into English. In-depth interviews took around 40–60 min each. Focus group discussions lasted about 60–90 min.

### Data analysis

Team-based thematic and logical analysis was conducted manually by five researchers. Two independent, multi-lingual researchers verified the transcriptions against audio recordings to ensure that translations were done correctly. Transcripts were read thoroughly by the team to document themes articulated directly by respondents (verbatim) or identified by the team during analysis discussions. Using structural and content coding techniques, the team developed a codebook that illustrated the final thematic observations. All in-depth interview and focus group transcripts were coded according to emerging patterns; these codes were then further refined through a series of iterative cycles used in team-based qualitative analysis. Coding was conducted by two members of the research team who then compared work for consistency, ensuring high inter-coder reliability.

### Ethical considerations

Ethical approval for this study was obtained from the Ethical Review Committee (ERC No# 2020–5316-14620) of the Aga Khan University, Karachi, Pakistan, before any data collection.

## Results

We conducted 60 in-depth interviews (IDIs) with parents/caregivers and seven focus group discussion sessions (FDGs) with healthcare workers (doctors, nurses, pharmacists, LHWs, etc.), with a total of 55 individual participants. Participant demographics are summarized for both healthcare workers and parents in Tables 1 and 2, respectively.

**Table 1:** Demographic characteristics of parents and caregivers

Parents/caregivers characteristics	(*N* = 60)
**Gender**	
Female	45(75%)
Male	15(25%)
**Median Age of Mother** **Median Age of Father**	29(48%) 33(52%)
**Language**	
Urdu	40(66%)
Sindhi	20(44%)
**Youngest child’s age (Years/Months)**	2 months to 1 year

**Table 2:** Demographic characteristics of Healthcare provider

Healthcare Providers Characteristics	(*N* = 55)
Doctors	3 (5%)
Vaccinators, Polio Supervisor and Polio UC-CSO	14 (26%)
LHWs, LHS, and LHV	34 (62%)
Pharmacist and Nurse	4 (7%)
**Work experience, years (median)**	19 years (Matiari) 12 years (Karachi) 10 years (CHC)
**Language**	Sindhi, Urdu (Matiari) Sindhi, Punjabi and Urdu (Karachi) Urdu, English (CHC)
**Education/Qualification level**	Range 10^th^ -16^th^ years

### Impact of the COVID-19 pandemic on childhood routine immunization: logistical barriers

#### Respondent perspective on importance of CRI during the pandemic

In both the rural and urban catchment areas, healthcare workers and parents perceived routine childhood immunizations as particularly important to build immunity against infectious diseases during the pandemic. In particular, healthcare workers viewed the pandemic as a demonstration of the need for additional measures to protect children from being infected with dangerous diseases such as COVID-19:

*“There is this growing awareness among parents that COVID-19 is here to stay and is a dark reality, but they (parents) should also take steps to protect their children from other life-threatening diseases for which EPI vaccines provide protection. So, for its (COVID-19) prevention, we need to get our kids vaccinated. (HCPs from CHC, Karachi)*

*“It (vaccine) boosts immunity by making protective antibodies against the virus and protect us from the virus (COVID-19)”* (HCPs from Matiari)

#### Logistical challenges to CRI during the pandemic

Parents shared their views on and experiences with several logistical facets of the vaccination system during the pandemic. Primarily, parents reported that the issuance of vaccination cards and the use of the electronic record system motivated them not to miss vaccination appointments scheduled for their children during the pandemic:

*“The whole record-keeping system is computerized. If I want a previous record, I can receive it even if I misplaced my child`s vaccination book. This is very convenient for me It`s written on the vaccination card. Those who don`t get their children vaccinated face many health issues” (Caregiver from AAG, Karachi)*

Many parents seemed to know which vaccination sites near them were open during the pandemic and reported feeling more relaxed attending the clinic in the COVID-19 period due to fewer patients and increased ease in performing routine childhood immunization services. Lower number of people at the center meant less chance of transmission as long as masking protocol was followed:

*“We heard (with COVID-19 disease) we will feel cold, have cough and fever, and runny nose and that we should wear face masks to prevent spreading it from one person to another” (Parent/caregiver from Matiari)*

#### Impact of the pandemic on outreach services

Another element of the Pakistani vaccination system are the immunization outreach services conducted by lady health workers (LHWs) in designated areas to track children who had missed vaccines and encourage their families to utilize immunization services. During the pandemic, LHWs and EPI vaccinators experienced heavier workloads and stricter performance accountability; similar outreach services in urban areas were limited in their community mobilization. In interviews, parents reported that they were not offered any door-to-door immunization services, nor did any HWs reach out to them to facilitate their children’s vaccination. However, healthcare workers interviewed did not report serious interruptions during the pandemic.

*“The EPI continued the same as before COVID-19 Pandemic, so we were not impacted much by the pandemic. We also conducted out-reach fields where parents used to visit to get their children vaccinated” (HCP from Matiari)*

#### Challenges to CRI demand and delivery during the pandemic

Despite disruptions like lockdowns, shortage of staff, fear, rumors in public, and limited vaccine supply, the EPI program continued to provide services. The WHO and CDC protective guidelines adopted by the government for EPI program included limited/relocated vaccination centers, shortened clinic hours, reduced patient capacity, masking, personal protective equipment (PPE) for the healthcare providers, social distancing, and shifting to or increasing outreach services like conducting door-to-door vaccination. Healthcare providers shared their experiences with challenges faced by the CRI delivery system, describing a lack of centers administering routine childhood vaccines, overcrowding in the existing open centers, long lines and waiting times, and reduced outreach programs in some areas:

*“The problem most people are currently facing with regard to routine childhood vaccine is very limited numbers of vaccination centers where they can get vaccinated. This creates long waiting time, and a person spends hours to get vaccinated. They must increase the vaccination sites to accommodate people who are willing to get vaccinated. Every EPI center must have the routine childhood vaccine available” (HCPs from Karachi)*

Parents described how difficult these logistical issues made it to vaccinate their child:

*“Reason for not getting vaccinations? because no one came here to vaccinate, neither did we go to the city for vaccination. Either we have to go to the hospital or where the child was delivered” (Parent/caregiver from Matiari)*

Despite efforts of LHWs to give BCG vaccines to every eligible child patient, rates of pentavalent, BCG, and pneumococcal vaccinations showed significant decline during the lockdown periods. Some community members were reluctant to visit EPI Clinics (center) as noted by healthcare providers and preferred home-based vaccination. Counseling parents to vaccinate their child was especially difficult, considering the public’s uncertainty about COVID-19 and the strong resistance of parents or caregivers to visit clinics during the pandemic. Even parents eager to get their child vaccinated struggled to do so: healthcare providers described how parents who had missed vaccinations due to lockdowns or fear of COVID-19 would arrive at the clinic, many months overdue for their appointments, and ask for the missed doses, compelling the staff to reconfigure suitable doses:

*“The main concern is that patients have missed essential vaccines during the last year due to COVID-19 fears. Now that they are visiting centers after a gap of 1 to 1.5 years, they are asking for those doses. It was tough to counsel and advise the parents in such a difficult scenario” (HCPs from AAG, Karachi).*

Finally, the rapidly changing context of COVID-19 safety and vaccination information made it difficult for both parents and healthcare workers. During interviews, healthcare workers reported:

*“CDC guidelines came gradually and not at once, so we were also very apprehensive regarding how to protect ourselves from getting infected in the initial days” (HCPs from Karachi)*

*“There is a change in work protocols too like we need to wear face masks, gowns and use hand sanitizers. During COVID pandemic these are also changes in normal work routine” (HCP from CHC, Karachi).*

In addition, rural parents shared that due to having limited knowledge about different diseases, instead of questioning or delaying, they follow their healthcare provider's decision on whether to vaccinate:

*“Doctors give the kids the routine vaccines. I am not sure what type of vaccines are given and for what diseases. We just rely on doctors for that”* (Parent from Matiari)

### Impact of the COVID-19 pandemic on childhood routine immunization

#### Social and emotional barriers

Most participants cited fear of COVID-19 virus as a cause of the substantial drop in delivery and utilization of immunization seen during the pandemic which causes social and emotional barriers as participants shared HCPs share low children visit at immunization clinics.

*“Towards the start of the pandemic, the patient inflow ratio at our center was very low. Ever since COVID-19 came, patients were generally very scared.” (HCPs from Karachi)*

*Many parents disagreed to get vaccinated at hospitals during the pandemic. Even when they were not feeling well, they avoided coming to hospitals and clinics as many rumors circulated in the communities then. People were scared and not going outside their homes” (HCPs from Karachi)*

Healthcare providers, although very motivated, expressed their fear of the disease and experiences of going about their daily routine in these challenging circumstances:

*“We were also fearful (of the virus) so how could we have asked parents to bring their children to centers during the pandemic”. (HCPs from Karachi)*

#### Impact of conspiracy theories on CRI and other preventive measures

The impact of conspiracy theories on community health initiatives (CRI) and other preventive measures in both urban and rural areas created potential to hinder the adoption of essential healthcare practices. For instance, parents from the rural site were skeptical of the reality of COVID-19: they questioned whether the pandemic was government propaganda or a conspiracy to divert the people’s attention from other issues such as poverty and global politics of domination:

*“Some parents were fearful and anxious about staff wearing masks and implementing social distancing protocols” (HCP from Matiari)*

*“People are reluctant to visit the center (because of masking and social distancing) and prefer visiting the field (outreach) more” (HCPs from Matiari)*

Heath workers also shared they engaged quite a bit with community members who denied the existence and severity of the COVID-19 virus. Healthcare providers shared their disappointment and viewed these community attitudes as not only a habitual source of frustration but also a disruption in service delivery:

*“Many people did not take COVID-19 Pandemic seriously and even joked (about it), that there is no COVID-19 Pandemic. When community members ridicule the disease, it decreases staff’s motivation to properly implement COVID-19 standard operating procedures (SOPs) in immunization clinics.” (HCP from Matiari)*

In rural communities, people continued to deny COVID-19 especially when it came to wearing a mask for social distancing:

*“Neither have we thought about it, nor have we ever seen, or even heard about any cases in our family or community – we have no such case” (parent from Matiari)*

### Acceptance and uptake of COVID-19 vaccination for adults

#### Healthcare worker awareness and knowledge of the COVID-19 vaccine

At the start of the pandemic, the Pakistan Ministry of Health made it mandatory for health care workers to be vaccinated as a priority group during the first phase of COVID-19 vaccinations:

*“In first phase, it is given to the front-line healthcare workers including doctors, nurses and paramedics staff” (HCPs from Karachi)*

In interviews, respondents shared that a lack of knowledge among HWs on the COVID-19 pandemic and the vaccines affected their confidence in communicating the importance of the vaccine to adults. This lack of communication ultimately affected vaccine acceptance and uptake among HWs, efforts which would have been more effective if HWs were more knowledgeable about the COVID-19 vaccination.

#### Healthcare worker concerns regarding the COVID-19 vaccine

A common concern among healthcare workers regarded the safety of being vaccinated during pregnancy, as a large majority of frontline community healthcare workers were female. In the urban areas, these HWs were able to discuss their concerns with senior medical doctors; however, LHWs in rural areas relied on government officials, who simply assigned them as ‘ineligible for the vaccine’ due to pregnancy:

*“I was informed that I am currently not eligible for the vaccine since I am pregnant therefore, I have not got it.” (LHW from Matiari) “After my concerns were clarified by a senior doctor, I decided to get my vaccine and today is my 2nd dose.” (LHV from AKU-CHC)*

Healthcare providers in urban areas were more aware of the vaccination-related information as they cited concerns regarding the storage temperature for the Pfizer vaccine in comparison to the Sinopharm vaccine in rollout efforts in Pakistan:

*“Pfizer vaccines require storage temperatures that cannot be maintained in Pakistan. Hence,we got Sinopharm vaccines and needs to be kept at a cold chain temperature of 2 to 8 Celsius” (HCP from Karachi)*

For healthcare providers, reasons for COVID-19 vaccine hesitancy mostly had to do with misconceptions about the pandemic, disbelief in the existence of the COVID-19 disease, and disbelief in the efficacy of the vaccine itself. Healthcare providers doubted the efficacy because they thought that the vaccine was ‘*rushed*,’ the ‘*trials were not completed properly*,’ and felt there was no surety of its efficacy without full knowledge of the side effects in humans. Urban healthcare providers were also of the view that, given these doubts, taking the vaccine should be a personal choice:

*“It was my own independent decision, so I got the COVID-19 vaccine for myself” (HCPs from Karachi)*

### Acceptability of a potential COVID-19 vaccine for children among HWs and parents/caregivers

Healthcare providers and parents seemed to be more open about the COVID-19 vaccine for children, as compared to the same vaccine for adults. The primary reason for this acceptance was the belief that children are at higher risk of exposure to infectious diseases in schools and other public places, and therefore should receive a protective vaccine. This data was collected when the trials on COVID-19 vaccine for children were close to the finish line.

Similarly, the parents and caregivers of children under the age of 5 years held the same views on COVID-19 vaccine for children as, “*it will develop immunity against COVID-19 infection*”. Parents were aware of and seemed excited about the on-going trails for COVID -19 vaccination for children at time. LHWS from all three sites strongly supported the free provision of COVID-19 vaccines for children whenever made available. However, they categorically advised ‘*only administrate after proper scientific studies have been conducted.’*

*“All COVID-19 vaccine trials in Pakistan and the rest of the world were done in adults above 18. So, scientists are not sure what effects this COVID vaccine will have on children. Research is* not yet complete” – (HCPs from Karachi).

*“Yes, I feel it should be available for children after proper research. It will protect children.” (Caregiver from Karachi)*

Other potential factors cited by parents in COVID-19 vaccine uptake in children included the cost of vaccines (free vaccines in government routine childhood immunization centers versus out-of-pocket payments in private centers), the availability and provision of COVID-19 vaccines EPI program, transport facilities to the centers, and nearby out-reach facilities.

### Should childhood COVID-19 vaccine be included in the EPI program?

While the majority of parents responded in favor of childhood COVID-19 vaccines to be included in the routine childhood immunization through EPI program platform in the future, some parents also expressed concern. One of the reasons was lack of information about these vaccines,

*“I have heard that there is a vaccine for protection against COVID-19 and I don`t know about benefits or harmful effects of COVID-19 vaccines” (Parent from Matiari)*

Another reason cited was perceived pain and side effects associated with childhood vaccines. For this reason, some parents suggested providing it separately ‘*not at the time as routine immunization schedule’* so should not be included in routine childhood immunization.

*“Yes! It should also be available for children. It should not be part of regular preventive vaccines (routine child immunization) as it will cause more pain in children. They already get other vaccines” (Caregiver from Karachi)*

*“But I feel Children’s COVID-19 vaccine should be given separately. Kids are small. When the child gets any routine childhood vaccine, he gets a fever. That`s why the COVID-19 vaccine should be provided separately from other routine vaccine” (Parent/caregiver from Matiari)*

These parents also felt that government should provide an option to the parents to select vaccines for their children in the future, especially for deadly diseases like COVID-19 for which,

*‘no one knows its efficacy so it should be provided to children after parental consent and not forced on them.’*

*“It should not be a part of RI and be separate from EPI. There are many vaccines (influenza) that are given separately. The same should be with children’s COVID-19 vaccine in the future. If parents feel that it has no severe side effects in children and if they want it, then they can get it for children. If not, they have a choice to refuse it. It should not be compulsory”- (Parent from Karachi)*

## Discussion

In this study, we explored the perceptions of COVID-19, COVID-19 vaccination, and the barriers to childhood routine immunization which emerged during the COVID-19 pandemic amongst HWs and parents/caregivers from both rural and urban settings in Pakistan. Our study is among the few from LMICs to qualitatively examine such perceptions and barriers, including preventative measures (social distancing, etc.) and parental concerns about vaccine safety and efficacy. We found that while both caregivers and healthcare providers were motivated to vaccinate children during the pandemic, the pandemic presented nuanced challenges to CRI and COVID-19 vaccine uptake during its early stages.

The unequal distribution and prioritization of COVID-19 vaccinations during the early stages of the pandemic contributed to the disparities in burden of disease that have been observed among different racial, ethnic, and socioeconomic groups in Pakistan and around the world [18–20]. The COVID-19 pandemic has led to system-wide interruptions in routine immunization programs globally, due to factors such as lockdowns, restrictions on movement, and concerns about exposure to the virus during clinic visits [21]. In this study, we explored the extent to which these kinds of disruption impacted routine immunization in Pakistan, and how the public perceived the potential integration of the COVID-19 vaccine into existing immunization programs. Our findings highlighted several factors that influenced adults' willingness to receive the COVID-19 vaccine such as availability, accessible through EPI program, awareness and counselling them regarding side vaccine safety. In addition to these logistical barriers, our study revealed a lack of awareness, knowledge, and trust in the vaccine, underlying a skepticism towards COVID-19 and a disbelief in the efficacy of the COVID-19 vaccine. These findings are similar to those presented in another study conducted in Pakistan by Qamer AM et al., which reported that an obstacle to the implementation of COVID-19 vaccine initiatives was the spread of falsehoods regarding the vaccine's biological consequences, notably the vaccine's alleged capacity to modify DNA and implant microchips to manipulate human behavior [22].

Understanding that barriers to vaccination in Pakistan include concerns about the safety and efficacy of the vaccine, a lack of trust in the healthcare system or government, and misinformation about the vaccine, will allow healthcare workers and policymakers to develop effective strategies to improve vaccine acceptance and uptake. These concerns are not solely related to the vaccination of adults: in the case of children, our respondents seemed to believe that vaccination was an important step in controlling the pandemic, but did raise concerns about vaccine safety and efficacy, especially given that children may have a different immune response to the vaccine than adults. These concerns are not unlike those reported in a similar study in Ghana, where parental vaccine hesitancy was linked to education levels, doubts about COVID-19 curability, and concerns about vaccine safety [23].

**Fig. 1:**
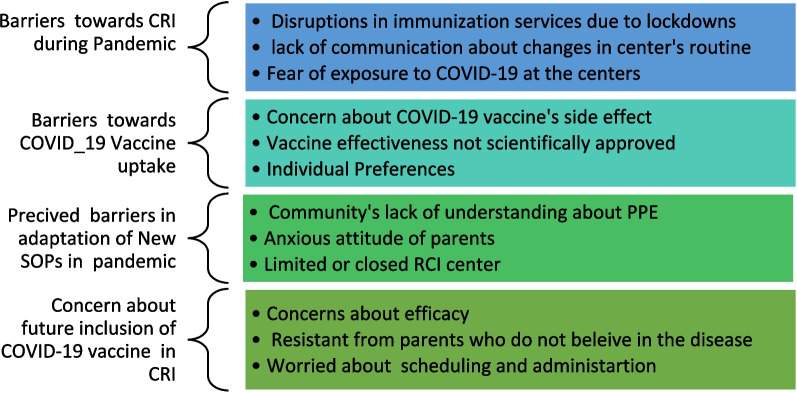
Healthcare providers views and experiences about barriers related to routine childhood immunization and COVID-19 vaccine hesitancy during pandemic in Pakistan

**Fig. 2:**
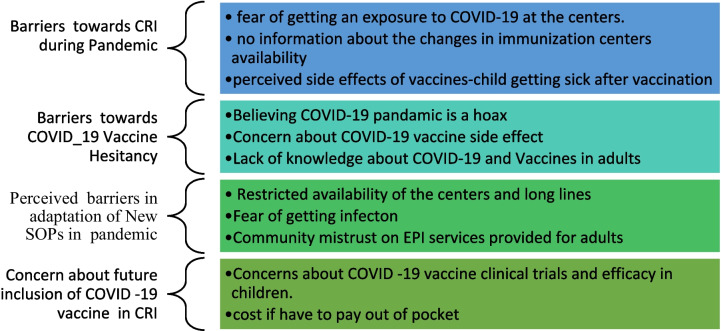
Parent’s/caregiver’s views and experiences about barriers related to routine childhood immunization and COVID-19 vaccine hesitancy during pandemic in Pakistan

For CRI, the barriers during pandemic were logistical, and included closed vaccination centers, drastic reduction in outreach programs, lack of information for the parents on vaccine availability, fear of pandemic, and lack of vaccine supply and healthcare staff. For COVID-19 vaccines, the challenges were more social in nature, including skepticism about the reality of the pandemic; fear around COVID-19; confusion over COVID-19 vaccines due to conflicting (or mis-or-dis) information from various sources, especially the rapid research and development of these vaccines; and general trust or mistrust of government systems (Refer to Fig. 1). However, when interviewed, community members were open to the possibility of incorporating COVID-19 vaccines for children in Pakistan’s Expanded Program for Immunization if required in the future (Refer to Fig. 2).

Our study provided insights regarding the potential for CRI outreach services to recuperate vaccination uptake during the pandemic. Many parents in urban, rural, and peri-urban areas indicated uncertainty and unprecedented dread during COVID-19 pandemic due being unable to vaccinate their children early in the pandemic (Refer to Figs. 1 and 2). Our study revealed that irrespective of the barriers, parents preferred CRI completion for their infants. These findings are similar to a study conducted in England by Bell et al. in which they found that 86% of parents got their children vaccinated according to scheduled vaccination time during the pandemic [24]. However, despite parents eagerness to vaccinate their children, vaccination rates did decline by more than 50% in both LMICs and developed countries due to substantial interruptions like those described here [9, 12, 13].

Lockdowns imposed by the government significantly impacted attendance at CRI facilities, and caregivers experienced transportation challenges in rural and peri-urban regions. These lockdowns coincided with the global drop in the number of children immunized by routine immunization programs, specifically, in the Central African Republic (CAR), Ghana, Guinea, and Senegal [25, 26]. However, despite the hurdles posed by the pandemic to regular childhood and COVID-19 vaccination programs, both caregivers and healthcare providers were motivated to vaccinate the children. This motivation was similar to that see in Southeastern Nigeria, where communities believed that the development and rollout of COVID-19 vaccines would help to end the pandemic and get people back into their daily routines, in good health [27].

Most HCPs expressed satisfaction about the safety and efficacy of COVID-19 vaccines available in Pakistan: this is crucial, as this trust in vaccines will ease doubts about vaccination. This finding was consistent with other studies in which positive sentiment towards vaccines was high among doctors and HCPs [28, 29]. In our study, we found that more responsibilities were assigned to healthcare workers and doctors to raise awareness and improve community advocacy campaigns, as well as to conduct regular visits without much training or incentives. Furthermore, CHWs played a critical part in immunization programs during COVID-19 to encourage parents and continued providing services during the pandemic and lockdowns [16]. Still, there were concerns that this might unnecessarily lead to an irrecoverable loss of trust in the EPI program in rural settings if any severe side effects or death occurred in any child receiving the COVID-19 vaccine in the future. These findings were similar to those of a study conducted by Peter G et al., in which many parents were cautious about COVID-19 immunization for themselves, which is strongly predictive of their apprehension about vaccinations for their children [30].

**Fig. 3:**
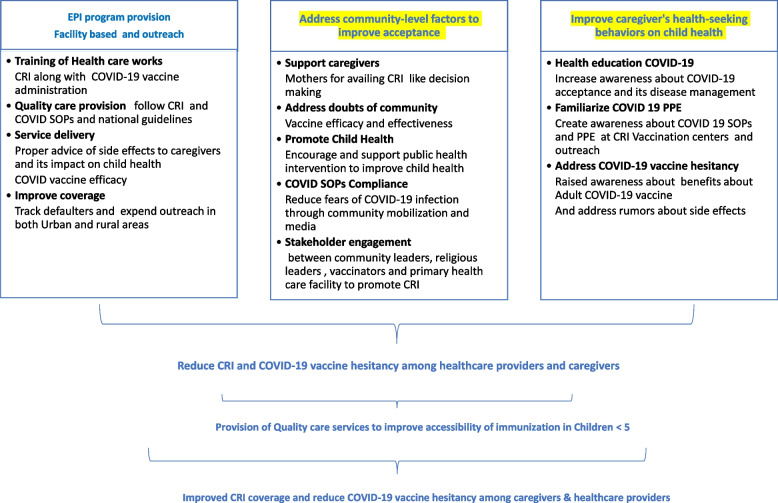
Framework to improve CRI coverage and reduce COVID-19 vaccine hesitancy among caregivers and healthcare providers

Lastly, the findings of our study sought to answer the question of whether parents would be willing to have their children vaccinated against COVID-19. According to our findings, HCPs believed that COVID-19 vaccine should be available for children since they are at a higher risk of exposure in schools and included as part expanded program of immunization. Similarly, parents preferred completion CRI for their infants despite the challenges of achieving this during the pandemic. Our study data will help mitigate the rising COVID-19 cases seen during different waves. The demand for children's COVID-19 vaccine within the framework of EPI in the future will further help in COVID-19 vaccine acceptance and compliance as favored by the HCPs in this study. Based on our findings, we propose a framework to address these issues (Refer to Fig. 3). This framework aims to improve routine immunization coverage and reduce vaccine hesitancy among healthcare providers and caregivers to improve child health outcomes, especially during the COVID-19 pandemic. It includes three major steps: providing EPI programs through facility-based and outreach services, addressing community-level factors that influence vaccine acceptance, and addressing caregiver health-seeking behaviors to improve child health outcomes. The framework is a comprehensive approach to improving immunization coverage and reducing vaccine hesitancy among healthcare providers and caregivers.

### Recommendations

Strategic approaches to deliver timely information and services are key components of increasing community confidence in vaccines. Catchup campaigns and future pandemic interventions should include information on alterations to the regular immunization system – for example, whether certain vaccine centers are open or not, and what alternative’s locations are available. We also recommend efforts to build resilience through systems-based interventions such as investing in surge capacity in the immunization workforce, which would allow the healthcare system to bounce back quickly after disruptions from initial pandemic waves.

Strong community outreach, including continued focus on educational initiatives, is also needed; particularly for COVID-19 vaccines for children (where recommended), health providers and other reliable sources of information may help to encourage parents who wish to "wait and see" to vaccinate their children. Effective preparedness measures are urgently needed to address the obstacles discussed in this paper and to improve and maintain robust immunization services during present and future pandemics.

### Study limitations

One significant limitation of the study was that the findings were derived from participants visiting PHCs run under private health entity (AKUH) opening timings, human resources, and budget restraints. Therefore, it might be harder to generalize the findings related to COVID-19 vaccine hesitancy to other groups with different social and cultural backgrounds, such as Western communities. Nevertheless, the research remains instrumental for further investigation by policymakers to cause a positive shift in people`s attitudes and practices regarding vaccine acceptance in the post-COVID-19 era.

## Conclusion

The disruptions to the Pakistani immunization system during the pandemic were not due to the lack of parental eagerness to vaccinate but rather to the rapidly changing context of the pandemic which made it difficult to provide services safely. Access issues and concerns about the safety of clinic visits also contributed to the uncertainty regarding availability of the vaccination services in the early part of the pandemic. Strategic approaches to delivering information and services promptly are a key component of increasing community confidence. For catchup campaigns and future pandemics, interventions should be focused on persuading people to vaccinate themselves and their children, but more so on building resilience through strategic system-based interventions, and strong community outreach.

## Data Availability

The datasets used and/or analyzed during the current study are available from the corresponding author on reasonable request.

## Abbreviations

AKUH: Aga Khan University Hospital
PHCs: Primary healthcare centers
EPI: Extended program of immunization
CRI: Childhood Routine Immunization
CSOs: Civil Society Organizations
BCG: Bacilli Calmette-Guerin
DPT3: Diphtheria, pertussis, and tetanus
LMICs: Lower-middle-income-countries
PCCHI: Pakistan CSOs Coalition for Health and Immunization
DHS: Demographic Health Survey
WHO: World Health Organization
EPI: Expanded Program for Immunization
IDIs: In-depth interviews
FGDs: Focus group discussions.
LHVs: Lady Health Visitors
LHWs: Lady Health workers
CHWs: Community Health Workers
HWs: Health workers
PHC: Primary Healthcare Centers
HDSS: Health Demographic Surveillance System
CHC: Community Health Center
ERC: Ethical Review Committee
